# Expression dynamics of phytochrome genes for the shade-avoidance response in densely direct-seeding rice

**DOI:** 10.3389/fpls.2022.1105882

**Published:** 2023-01-18

**Authors:** Yongtao Cui, Minhua Zhu, Jian Song, Honghuan Fan, Xiaozheng Xu, Jiayan Wu, Longbiao Guo, Jianjun Wang

**Affiliations:** ^1^ Institute of Crops and Nuclear Technology Utilization, Zhejiang Academy of Agricultural Sciences, Hangzhou, China; ^2^ College of Landscape and Architecture, Zhejiang A&F University, Hangzhou, China; ^3^ College of Advanced Agriculture Sciences, Zhejiang A&F University, Hangzhou, China; ^4^ State Key Laboratory of Rice Biology, China National Rice Research Institute, Hangzhou, China

**Keywords:** oryza sativa. L, densely direct seeding, phytochrome, miR156, miR172, early flowering

## Abstract

Because of labor shortages or resource scarcity, direct seeding is the preferred method for rice (*Oryza sativa*. L) cultivation, and it necessitates direct seeding at the current density. In this study, two density of direct seeding with high and normal density were selected to identify the genes involved in shade-avoidance syndrome. Phenotypic and gene expression analysis showed that densely direct seeding (DDS) causes a set of acclimation responses that either induce shade avoidance or toleration. When compared to normal direct seeding (NDS), plants cultivated by DDS exhibit constitutive shade-avoidance syndrome (SAS), in which the accompanying solar radiation drops rapidly from the middle leaf to the base leaf during flowering. Simulation of shade causes rapid reduction in phytochrome gene expression, changes in the expression of multiple *miR156* or *miR172* genes and photoperiod-related genes, all of which leads to early flowering and alterations in the plant architecture. Furthermore, DDS causes senescence by downregulating the expression of chloroplast synthesis-related genes throughout almost the entire stage. Our findings revealed that DDS is linked to SAS, which can be employed to breed density-tolerant rice varieties more easily and widely.

## 1 Introduction

The world’s population is expected to reach 9.3 billion by 2050, and food security is becoming an increasingly serious global issue (Warnasooriya and Brutnell, 2014). Rice is a common staple food (Chen et al., 2022). Direct seeding of rice has become popular in China during the last few decades (Farooq et al., 2011; Zhou et al., 2019). Furthermore, improving rice yields per unit land area by employing an optimal density of direct seeding is a viable option. Density tolerant rice varieties have greater vigor, allowing the possibility of direct sowing and increasing the yield per unit land area. However, densely direct seeding (DDS) can result in competition by neighboring plants, limiting rice production. Shade-avoidance syndrome (SAS) is the name given to this phenomenon in *Arabidopsis thaliana*. Rice plants change their type to compete with their neighbors for limited resources in order to optimize their life cycle when SAS occurs (Xie et al., 2017). Elucidating the mechanism that regulates SAS in rice can aid scientists in developing new high density-tolerant rice cultivars, making direct-seeding procedures for rice cultivation more popular as a result.

Light is an essential environmental signal for plants (Takanoa et al., 2009). Phytochromes are believed to be the primary mediators of the SAS (Xie et al., 2017). The *phytochrome-PHYTOCHROME-INTERACTING FACTORS(PIFs)* signaling pathway and the *MIR156-SQUAMOSA PROMOTER BINDING PROTEIN-LIKE (SPL)* regulatory module regulates phytochrome-mediated SAS responses in *Arabidopsis thaliana* (Xie et al., 2017). Five *PHY* genes exist in *Arabidopsis thaliana*, i.e. *PHYA* to *PHYE*, with only *PHYB* playing a substantial role in SAS (Xie et al., 2017). Differently, rice has only three genes: *PHYA*, *PHYB*, and *PHYC* (Takanoa et al., 2009). Rice *PHYA* has been reported to govern photomorphogenesis in two separate modes of photoperception (R/FR), while *PHYC* is involved in the photoperception of FR for deetiolation, according to physiological investigations of *PHYA* mutants (Takano et al., 2005; Takanoa et al., 2009). Furthermore, under long-day (LD) conditions, both *PHYA* and *PHYB* single mutants are believed to lead to early flowering (Ishikawa et al., 2011; Osugi et al., 2011), suggesting that each rice phytochrome plays a unique role in regulating key daylength recognition gene expression. In plants, *miR156* works as a master regulator for a variety of biological processes, including SAS (Huijser and Schmid, 2011). The 11 *miR156* genes (pre-*miR156a* to *pre-miR156l*) are found in the rice genome, and are involved in regulating critical processes such as seed dormancy, seed germination, plant shoot architecture, and grain size (Miao et al., 2019). During vegetative development in plants, *miR156* and *miR172* act as antagonistic agents (Chuck et al., 2007; Wang et al., 2009; Wu et al., 2009). *The miR156-SPL* and *miR172-APETALA2 (AP2)* modules play a crucial role in flowering as critical regulators of the time of the vegetative to reproductive phase transition (Wang et al., 2009; Wu et al., 2009; Cui, 2020).

SAS has been shown to cause early flowering in previous research (Casal, 2012). Rice is a common short day (SD) crop (Chen et al., 2022). It has evolved two flowering pathways: (the *Hd1*, *Ghd7*, *DTH8*, *OsPRR37*)-*Ehd1-Hd3a/RFT1* long day (LD) flowering suppression pathway and the *OsGI-Hd1-Hd3a/RFT1* SD flowering-promotion pathway (Chen et al., 2022). The downstream core flowering regulatory genes *Hd3a* and *RICE FLOWERING LOCUS T1 (RFT1)* are two florigen genes (Zhao et al., 2015; Chen et al., 2022). In both LD and SD, *Ehd1* encodes a B-type response regulator that promotes heading by upregulating the expression of *Hd3a* and *RFT1* (Zhao et al., 2015; Cho et al., 2016). *RID1/Ehd2/OsID1, SE5, OsMADS51* and other upstream genes positively regulate *Ehd1* (Kim et al., 2007; Wu et al., 2008; Andres et al., 2009), whereas *Heading Date 1* (*Hd1*), *Ghd7*, *COL4*, and *DTH8/Ghd8/Hd5* negatively regulate it (Yano et al., 2000; Xue et al., 2008; Lee et al., 2010; Wei et al., 2010; Chen et al., 2022). *Hd1* has a dual function that suppresses heading in LD and promotes it in SD (Yano et al., 2000). The status of other essential flowering regulatory genes *Ghd7, Hd2*, and *DTH8* are required for *Hd1* to reduce heading in LD (Zhang et al., 2017; Zong et al., 2021). *SE5* is involved in the biosynthesis of phytochrome chromophore (Andres et al., 2009). By affecting both the *Hd1* and *Ehd1* flowering pathways, the *se5* mutant exhibited early heading under both SD and LD conditions. The *AP2* genes, which contain the *miR172* target site, act as *Ehd1* repressors (Lee and An, 2012; Lee et al., 2014).

The mechanism of SAS in plants is conservative. Significant progress has been made in understanding shade avoidance during the last decade (Casal, 2012; Ciolfi et al., 2013; Xie et al., 2017; Liu et al., 2019; Paulisic et al., 2021). Rice is important for agriculture and the economy. Direct-seeding must be developed in order to increase grain yield while reducing labor and resource costs. We investigated the shade-avoidance response at various times in this study using gene expression under normal direct seeding (NDS) and densely direct seeding (DDS) conditions. Dynamic expression of the genes involved in the *phy-miR156s* signaling pathway and the photoperiodic flowering pathway were also investigated in this study because *miR156s* mediates phytochrome-mediated SAS responses. Furthermore, we also studied the dynamic expression of chlorophyll metabolism. Based on these findings, we argue that altering the *phy-miR156s* signaling pathway is an approach to adapt direct-seeding to a high-density environment.

## 2 Materials and methods

### 2.1 Plant materials and growth conditions

The rice cultivars Nipponbare and four mutants (i.e., *phyb、OEmiR156b/c、ipa1-2D、Ostb1*) were chosen for this study. Seed dormancy was broken by exposing the seeds to 45°C for 3 days, followed by pre-germination then sowing the seedlings in a nursery in a 2 m by 10 m plot. DDS was implemented by planting two plants with no more than a distance of 1 cm between each plant in a row and 1 cm between the rows. NDS was implemented by planting two plants with a distance of 20 cm between each plant in a row and 20 cm between the rows. Both plots received the same fertilizer management.

### 2.2 Agronomic trait measurements

Once the plants achieved maturity, the primary agronomic traits (i.e., plant height, tiller, leaf length, leaf breadth, spikelet length, the number of primary branches, and the seed-setting rate) were ascertained. Data from three biological replicates were compiled for each trait. For each attribute, data from three biological replicates was compiled.

### 2.3 Small RNA blot analysis

Total RNA was isolated from rice leaves using the TRIzol reagent (Invitrogen); 10 µg of total RNA were fractionated on 17% polyacrylamide gels under denaturing conditions (i.e., 7 M urea). Blots were hybridized using digoxigenin end-labeled LNA oligonucleotides (Exiqon, Vedbaek, Denmark) complementary to the *miR156* or *miR172* sequences. The assay was conducted as previously described (Guo et al., 2016). The following DNA oligonucleotides were used: miR156 probe:5’-GTGCTCACTCTCTTCTGTCA-3’, miR172 probe: 5’-GCAGCACCATCAAGATTCAC - 3’, U6 probe: 5’- TGTATCGTTCCAATTTTATCGGATGT - 3’.

### 2.4 RNA Extraction and RT-PCR

Total RNA was extracted from rice leaves using the TRIzol reagent (Invitrogen), and cDNA was made using 1 µL of RNA and ReverTra Ace qPCR RT Master Mix with gDNA Remover (TOYOBO), as directed by the manufacturer. Each cDNA replicate was subjected to qPCR, and all samples were processed in duplicate. A Power SYBR Green PCR Master Mix kit was used for the quantitative real-time PCR investigations (Applied Biosystems). PCR conditions were 95 °C for 10 minutes, 40 cycles of 95 °C for 15 seconds, 60 °C for 1 minute, and a final melt curve step from 60 °C to 95 °C. The average expression ratios were obtained from the equation 2^-△△CT^, where △△CT represents △CT (gene of interest in stage evaluated)-△CT (gene of interest at control stage), according to the protocol reported by Cui (Cui et.al., 2020).

### 2.5 Radiation intensity measurement

At noontime, a spectrograph (Spectrometer MAYA 2000, 830 Douglas Ave., Dunedin, FL, USA) was used to measure the absolute visible light radiation intensity at the flag leaf, the middle leaf and the base leaf from NDS and DDS plants during the flowering period. Three biological replicates were used.

### 2.6 Chlorophyll content measurement

The content of chlorophyll was determined using Xu’s technique (Xu et al., 2017). Cut leaves (0.2 g fresh weight) were soaked in 10 mL ethanol for 48 h in the dark. The remaining plant debris was removed by centrifugation. A UV-VIS spectrophotometer (Shimadzu, UV-2600, Japan) was used to analyze the supernatants at 663, 645, and 470 nm, respectively.

### 2.7 Statistical analysis

Based on three biological replicates, the data were expressed as the mean values with the standard deviation (SD). Student’s unpaired t test and Duncan’s multiple range test were used to determine statistical significance. Probability values of 5% were considered statistically significant; a single asterisk (*) and a double asterisk (**) denote statistical significance at the levels of 5% and 1%, respectively. The methodologies used in this study for the statistical analysis of the gene relative expression levels and the agronomic traits are all described above.

## 3 Results

### 3.1 Phenotypic and agronomic traits analysis

**Figure 1:**
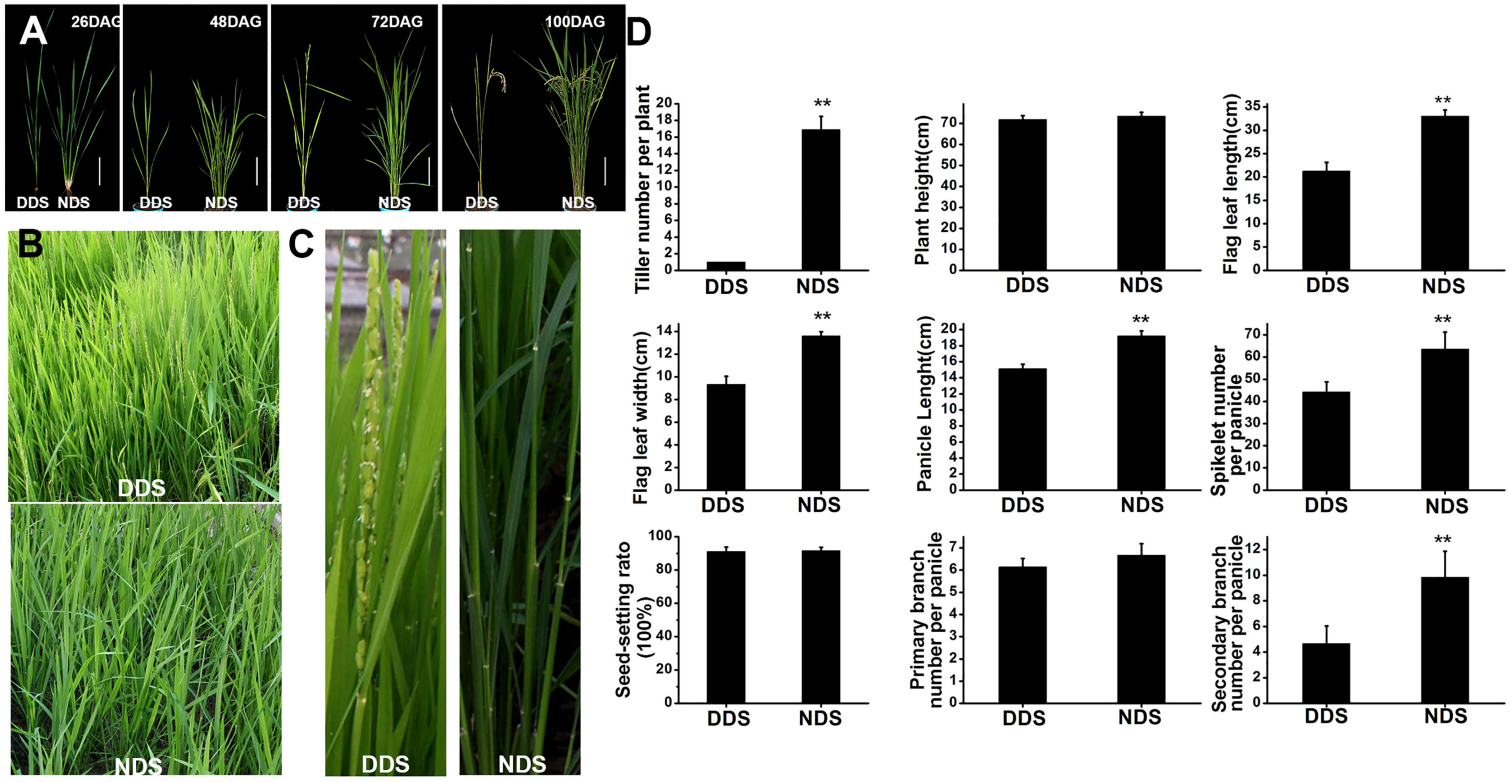
DDS plants exhibit constitutive SAS. **(A)** Comparison of the tiller at different stages under DDS or NDS conditions. 26, 48, 72, 100 DAG treatment were photographed. Bar = 15 cm. **(B, C)** Earlier heading dates and yellow leaves were observed in DDS at 72 DAG. **(D)** Agronomic traits in these plants. Data represent the mean ± SD of three biological replicates (Student’s t test: **P ≤ 0.01).

Shade-avoidance responses have previously been shown to include increased leaf angles to the horizontal, reduced branching, reduced leaf blade area, and early flowering (Casal, 2012). DDS, on the other hand, has consistently shown shade-avoidance traits throughout the life of the plant. Tiller number, plant height, flag leaf length and width, panicle length, grain number per panicle, seed-setting rate, and primary and secondary branch length and width were all measured. DDS plants have no or very little tiller during their lives (approximately only one tiller in total) (**Figures 1A, D**). In comparison to NDS plants, DDS plants had just 64% flag leaf length and 69% flag leaf width on average (**Figures 1B-D**). Furthermore, DDS plants exhibited early flowering and a short panicle length (~22% shorter than NDS), resulting in a significant reduction in grain number per panicle (~70% of NDS), primary branch (~92% of NDS), and secondary branch (~47% percent of NDS). However, the agronomic parameters (i.e., plant height and the seed-setting rate) did not differ substantially between DDS and NDS plants (**Figure 1**) (**Figures 1B-D**). Additionally, DDS plants displayed senescence. These observations indicate that DDS plants exhibited typical shade-avoidance traits in both the vegetative and adult stages.

### 3.2 Absolute visible light radiation intensity declined under DDS

**Figure 2:**
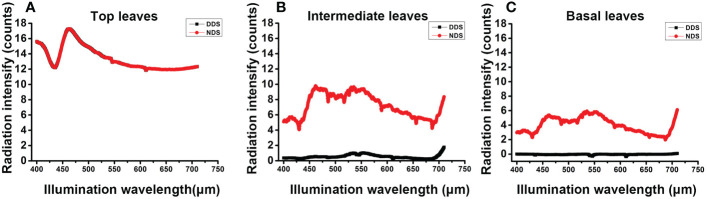
Radiation intensity declined under DDS. **(A)** The radiation intensity showed no difference at the top leaves at 72 DAG. **(B)** The radiation intensity was reduced in the DDS plants in intermediate leaves at 72 DAG. **(C)** The radiation intensity was reduced in the DDS basal leaves at 72 DAG. Data were measure for at least three biological replicates.

DDS permits plants to compete for limited resources with their neighbors (Ciolfi et al., 2013). We measured the radiation intensity at various leaves to determine the absolute visible light radiation intensity (i.e., the flag leaf, middle leaf and the base leaf). The radiation intensity at the flag leaf did not differ significantly between the DDS and the NDS plants at the flowering stage (**Figure 2**), however, the radiation intensity at the intermediate leaf and the base leaf was dramatically reduced in the DDS plants (**Figure 2B**). These results suggest that the normal R:FR ratio conditions have been altered, which simulated shade under DDS conditions.

### 3.3 The transcript levels of *PHYA*, *PHYB*, *PHYC* decreased under DDS conditions

**Figure 3:**
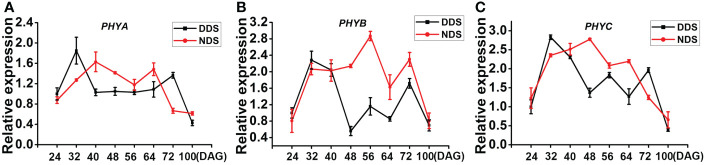
The transcript levels of *PHYA*, *PHYB*, *PHYC* decreased under DDS conditions. **(A-C)** The qRT-PCR analysis of temporal expression in the whole plants *PHYA*, *PHYB*, *PHYC* between DDS and NDS at 24、32、40、56、64、72、100 DAG. Error bars represent mean ± SD (n = 3) where the different letters indicate statistical differences according to Duncan’s multiple range test (p ≤ 0.05).

Phytochromes, which mediate SAS in plants, primarily respond to red (R)/far-red light (FR). We discovered that at 40, 48, 56, and 64 DAG under DDS conditions, the expression of the rice phytochrome genes *PHYA*, *PHYB*, and *PHYC* was down-regulated, indicating that the DDS plants had high overshadow. Only few differences were identified at 24 and 32 DAG, when both DDS and NDS plants were in the juvenile-to-adult stage (**Figure 3**). The DDS plants became a little flattened around 100 DAG, which slowed down the neighbors’ competition. We thus concluded that red (R)/far-red light (FR) of *PHYA/B/C* were largely weakened under DDS conditions.

### 3.4 The transcript levels of *miR156s* and *miR172s* increase under DDS

**Figure 4:**
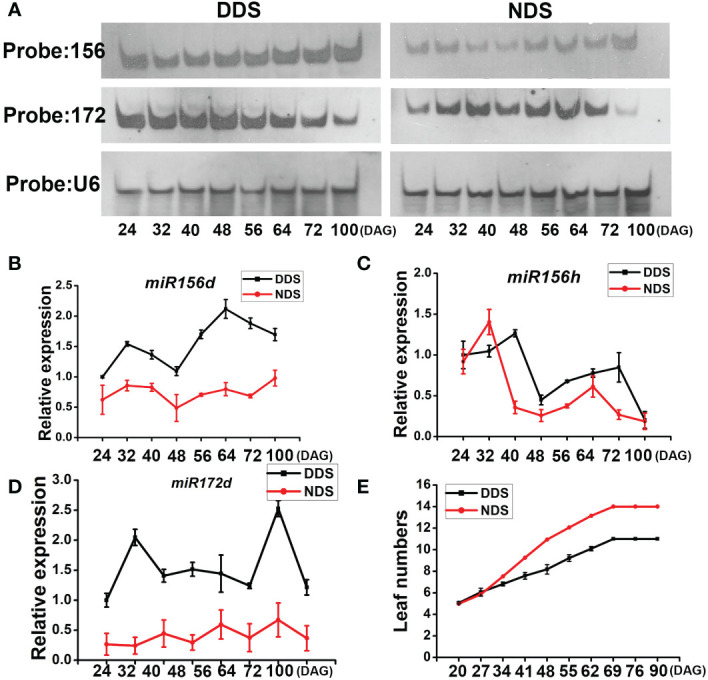
Ectopic expression of *miR156s* and *miR172s* in DDS plants. **(A)** Northern blot of *miR156s* and *miR172s* expression levels in whole plants at either the adult or juvenile stages between DDS and NDS. The RNA samples in each lane were extracted from whole plants. U6 was used as a loading control. **(B, C)** A Quantitative reverse transcription polymerase chain reaction (qRT-PCR) analysis of *pri-miR156d/h* expression between DDS and NDS at 24、32、40、56、64、72、100 DAG. **(D)** A qRT-PCR analysis of temporal expression of *pri-miR172d* between DDS and NDS at 24、32、40、56、64、72、100 DAG. **(E)** A shorter plastochron and fewer leaves were observed for DDS compared to NDS. Error bars represent mean ± SD (n = 3) where the different letters indicate statistical differences by Duncan’s multiple range test (p<0.05).

In plants, *miR156* is a conserved regulator of developmental time. Overexpression of rice *miR156B/C* resulted in more tillers, shorter and narrower leaves, a longer duration of the expression of juvenile vegetative features, and later flowering (Cui 2020). We looked at the expression of leaves under both conditions to see if *miR156s* played a role in the response to DDS. We employed a northern blot to detect the level under both conditions to measure the total level of *miR156s in vivo* and evaluate the dynamic expression of *miR156s*. At 24 DAG, 32 DAG, 40 DAG, 48 DAG, 56 DAG, 64 DAG, 72 DAG, and 100 DAG, RNA samples were collected from the leaves of entire plants. Under DDS conditions, we saw a higher concentration of *miR156* transcripts throughout the stage than under NDS conditions (**Figure 4**). On the other hand, SAS caused *miR156* to be downregulated in *Arabidopsis*. Th *miR156* transcript level dropped at first and later increased under both conditions. A quantitative reverse transcriptase RT-PCR experiment showed that the primary transcripts of *miR156D* and *miR156H* were clearly enhanced under DDS conditions (**Figure 4B**). During vegetative development in plants, *miR156* and *miR172* act as antagonistic agents (Chuck et al., 2007; Wang et al., 2009; Wu et al., 2009), so we looked at the levels of *miR172s*. In this study, the expression of *miR172s* steadily decreased in the DDS plants during shoot growth and maturation, while it gradually declined and then sharply decreased in the NDS plants at 100DAG (**Figure 4A**). DDS plants produced 10.2 leaves on average during their entire life cycle, 3-4 leaves less than the NDS plants (**Figure 4**). When compared to NDS, DDS heading dates were 4 days earlier. These findings reveal that under DDS conditions, both *miR156* and *miR172* were misregulated, resulting in early flowering, reduced organ size, and narrow leaves, and the DDS plants were more sensitive compared with the NDS plants.

### 3.5 DDS show early flowering

**Figure 5:**
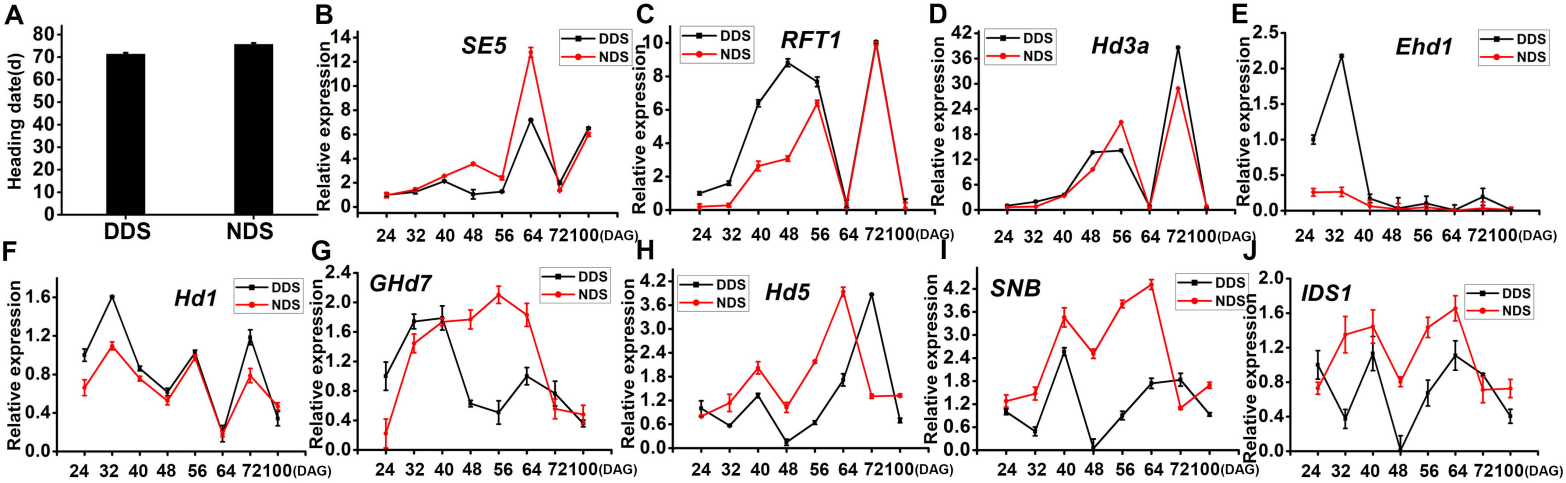
DDS showed early flowering and HD related genes. **(A)** The heading dates of NDS plants were delayed by 4 d, compared with the DDS. **(B-J)** qRT-PCR analysis of temporal expression in the whole plant of *SE5, RFT1, Hd3a, Ehd1, Hd1, Ghd7, Hd5, SNB, IDS1* between DDS and NDS at 24、32、40、56、64、72、100 DAG. Error bars represent mean ± SD (n = 3) where the different letters indicate statistical differences by Duncan’s multiple range test (p ≤ 0.05).

Rice is a typical SD plant with a strong connection to phytochromes. Compared to NDS, the DDS heading dates were 4 days ahead (**Figure 5**). Since the expression of *PHYA*, *PHYB*, and *PHYC* decreases after 40 DAG, this study focused on the flowering regulatory genes to study phytochrome-related responses. Under DDS conditions, the expression of *SE5* decreased from 48 DAG to 64 DAG (**Figure 5**). The downstream genes in the flowering pathway, *RFT1* and *Hd3a*, showed identical expression patterns under both LD and SD conditions. In this study, *RFT1* and *Hd3a* expression remained low for LD, but was dramatically up-regulated for SD under both DDS and NDS conditions (**Figure 5C**). In the LD state, however, *RFT1* expression was higher for DDS than NDS (**Figure 5**). Despite the fact that *Hd3a* expression did not differ considerably, there was a modest upregulation at 48 DAG (**Figure 5**). *Early heading date 1* (*Ehd1*) integrates many upstream regulatory signals to control the expression of the two florigen genes *Hd3a* and *RFT1*. For DDS, *Ehd1* showed much higher expression than NDS at 24 and 32 DAG, but only small differences at subsequent stages (**Figure 5**). Heading date *1* (*Hd1*) is a key gene in rice for flowering regulation; both DDS and NDS showed comparable tendencies throughout the stage, with the exception of overexpression at 24 and 32 DAG (**Figure 5**). In LD, *Ghd7* acts as a heading repressor. *Ghd7* showed an opposite expression trend after 40 DAG (**Figure 5**). *Hd5* expression was equivalent for DDS and NDS, but higher for DDS at 32-64 DAG (**Figure 5**). *SNB* and *IDS1* are two *AP2* transcription factors that function downstream of *miR172*. At 32 to 64 DAG, both *SNB* and *IDS1* expression were increased (**Figure 5I**). These observations indicate that a gradual alteration of the expression of *miR156* and *miR172* results in changes in developmental phase transitions, leading early flowering under DDS conditions.

### 3.6 Relative expression of genes related to chloroplast development in DDS

**Figure 6:**
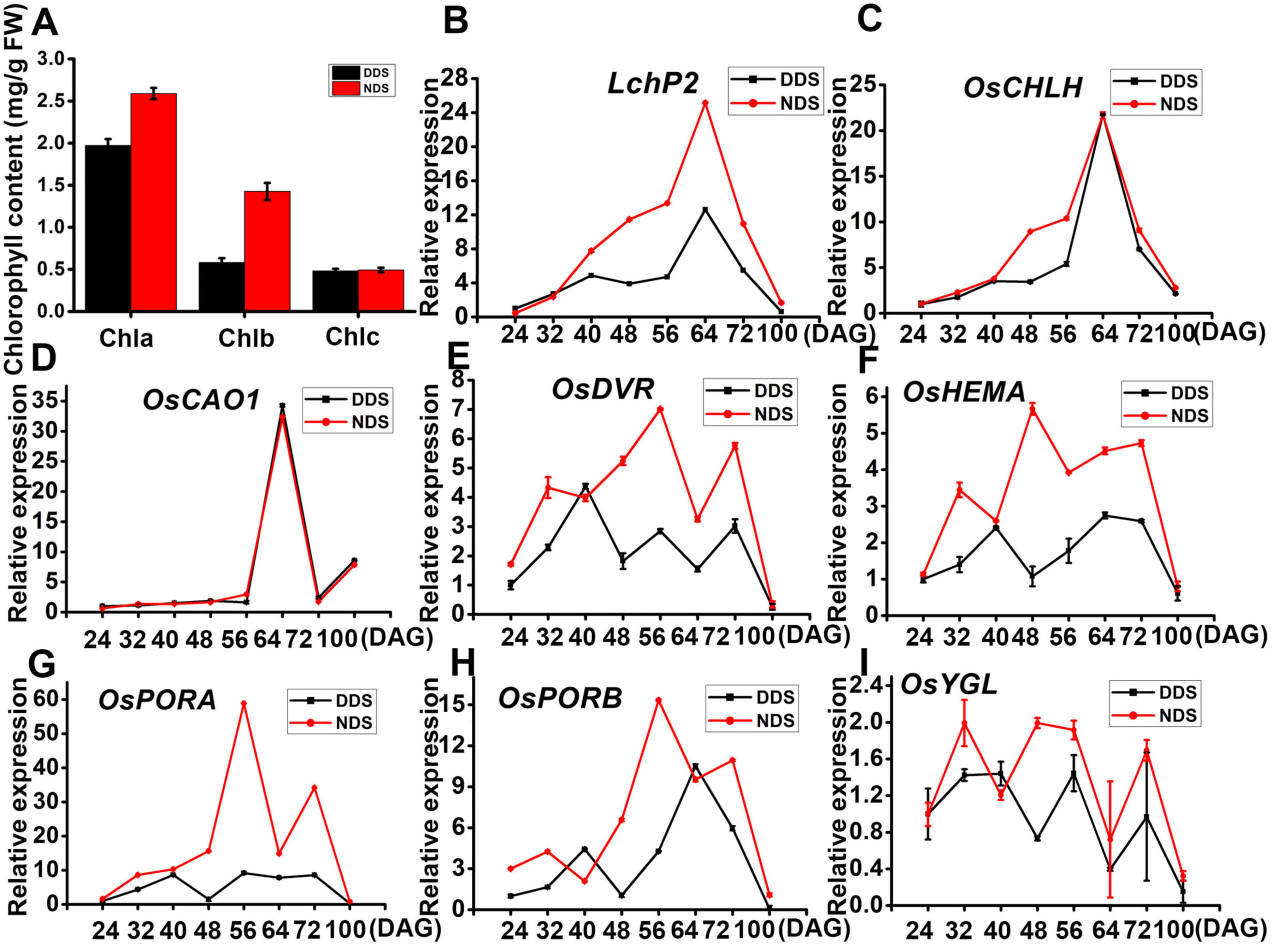
Relative expression of genes related to chloroplast development in leaves from DDS and NDS. **(A)** Content of photosynthetic pigments in flag leaves from DDS and NDS plants. **(B-I)** A qRT-PCR analysis of temporal expression in the whole plant with genes associated with chlorophyll synthesis (i.e., *LchP2, OsCHLH, OsCAO1,OsDVR, OsHEMA, OsPORA, OsPORB*, and *OsYGL*) between DDS and NDS plants at 24、32、40、56、64、72、100 DAG. Error bars represent mean ± SD (n = 3) where the different letters indicate statistical differences according to Duncan’s multiple range test (p ≤ 0.05).

Rice exhibited early flowering and senescence under DDS conditions. Chlorophyll metabolism and chloroplast formation are linked to senescence, so we looked at the transcript abundance of genes involved in chlorophyll synthesis and chloroplast development for both DDS and NDS. Initially, we determined the levels of the Chl a, Chl b, and carotenoid (Car) pigments for DDS and NDS flag leaves. We discovered that the content of Chl a and Chl b decreased dramatically (by 24% and 41%, respectively, compared to NDS) (**Figure 6**). Except for *OsCAO*, genes involved in chlorophyll synthesis (i.e., *LchP2, OsCHLH, OsDVR, OsHEMA, OsPORA, OsPORB*, and *OsYGL*) were considerably down-regulated under DDS compared to NDS conditions (**Figures 6B–I**). These results suggest that chloroplast biogenesis and development were seriously affected by DDS.

### 3.7 The expression of several *SPLs* were repressed in DDS plants

**Figure 7:**
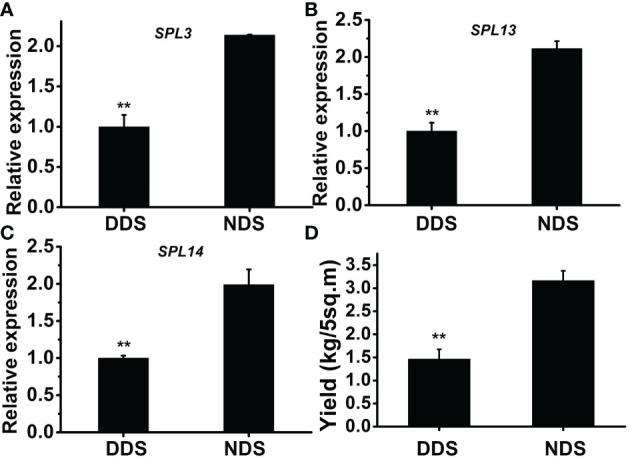
The theoretical yield was reduced in DDS plants. **(A-C)** A qRT-PCR analysis of temporal expression in the whole plant containing *SPL3, SPL13, SPL14* genes. **(D)** The theoretical yields were estimated. Data represent the mean ± SD of three biological replicates (Student’s t test: **P ≤ 0.01).

**Figure 8:**
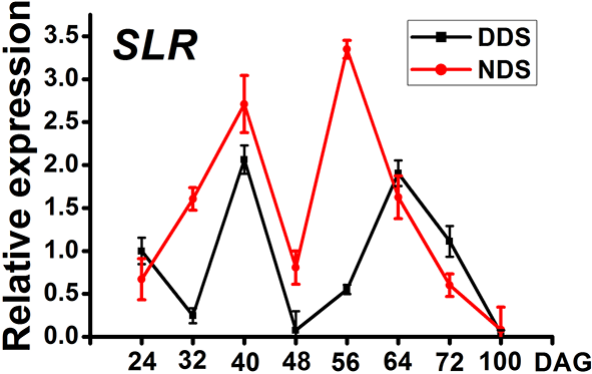
A qRT-PCR analysis of the temporal expression in the whole plants containing *SLR* between DDS and NDS at 24、32、40、56、64、72、100 DAG. Error bars represent mean ± SD (n = 3) where the different letters indicate statistical differences by Duncan’s multiple range test (p ≤ 0.05).

*SPL* genes are regulated by *miR156* and play a role in a variety of developmental processes. In *Arabidopsis thaliana*, active *SPL* genes control various morphological changes linked to shade avoidance responses. In DDS plants at 70 DAG, the expression of *OsSPL3*, *OsSPL13*, and *OsSPL14* decreased (**Figures 7A–C**). Comparing DDS to NDS, the potential yield was lower for DDS plants (**Figure 7**). In *Arabidopsis*, a link between gibberellin-mediated and *miR156/SPL* module-mediated flowering pathways has been established (Yu et al., 2012).In order to check whether DELLA protein SLR1(slender rice 1) was regulated by *miR156/SPL* genes, we analyzed *SLR*. The expression of *SLR* was decreased from 32 to 56 DAG (**Figure 8**). Taken together, these results suggest a link between of *OsSPLs* and *SLR* in DDS leading to fewer tillers.

## 4 Discussion

The *phytochrome-miR156* pathway regulates plant responses to SAS, allowing for rapid reconfiguration of the plant body while avoiding an unfavorable reaction to low R/FR (Xie et al., 2017). As a result, it has been widely assumed that plants have evolved the SAS during crop domestication and genetic improvement. Deciphering the mechanism that modulates SAS in DDS would thus aid scientists in the development of new density-tolerant cultivars for improved agronomic performance.

DDS causes a plant architecture phenotype in *Arabidopsis* similar to SAS (Xie et al., 2017). The majority of thorough SAS investigations have been in *Arabidopsis thaliana* (Casal, 2012; Xie et al., 2017; Liu et al., 2019; Paulisic et al., 2021). In response to simulated shade, a collection of signaling integrators between light and other signals has been shown. During SAS, inactivated *phyB* causes a rapid buildup of PIF proteins that bind directly to the promoters of numerous *miR156* genes, preventing the *miR156s* from inhibiting their target *SPL* genes. In this study, the dynamic expression of genes involved in the *phytochromes-miR156s/miR172s* or other signaling pathways were investigated using DDS. Fewer tillers, decreases in leaf length, width, grain number per panicle, and early flowering are all classic shade-avoidance responses induced by high density. These responses may have a favorable or negative impact on rice yield. The lower sunlight intensity in middle and base leaves under DDS conditions repressed the expression of rice phytochrome genes *PHYA, PHYB*, and *PHYC*, particularly *PHYB*. Phytochrome genes are thought to be entirely responsible for the perception of red and far-red light. The genes *miR156* and *miR172* are antagonistic toward vegetative development in plants. SAS inhibited the expression of *miR156s* in *Arabidopsis*. On the other hand, *miR156s/miR172s* were highly expressed under DDS conditions, mimicking the typical phenotype of *OEmiR156* (i.e., short and narrow leaves) (Cui et al., 2020). The difference in the expression of *miR156s/miR172s* between Arabidopsis and rice may due to treatment differences. In *Arabidopsis*, only the EOD-FR treatment was adopted to mimic shady conditions (Xie et al., 2017). However, under DDS conditions, rice plants might compete with their neighbors not only for light but also for the space necessary to complete their life cycle. Our findings suggest that DDS may have other effects in addition to SAS. The gene *miR156* regulates a variety of morphological changes linked with shade-avoidance responses by acting as a master upstream regulator of numerous genes such as *SPLs*. The decrease of yield is caused by the downregulation of *SPL3, SPL13*, and *SPL14* under DDS conditions, which causes a variety of morphological abnormalities. In *Arabidopsis*, a link between gibberellin-mediated and *miR156/SPL* module-mediated flowering pathways has been established (Yu et al., 2012). DELLAs interact with multiple flowering activators and repressors, resulting in late flowering phenotypes (Feng et al., 2008; Yu et al., 2012). Unlike Arabidopsis, which contains five DELLA genes, rice has only one, i.e. SLR1. Similar to the situation in Arabidopsis, *SLR* is also involved in the *Ehd1-Hd3a/RFT1* flowering pathway, resulting in late flowering (Zhang et al., 2022). Furthermore, the rice DELLA protein SLR1 interacts with MOC1 to regulate the tiller number (Liao et al., 2019). In this study, the decreased expression of *SLR* under DDS implies that GA is also involved in *miR156/SPL* module-mediated flowering pathways (**Figure 8**).

**Figure 9:**
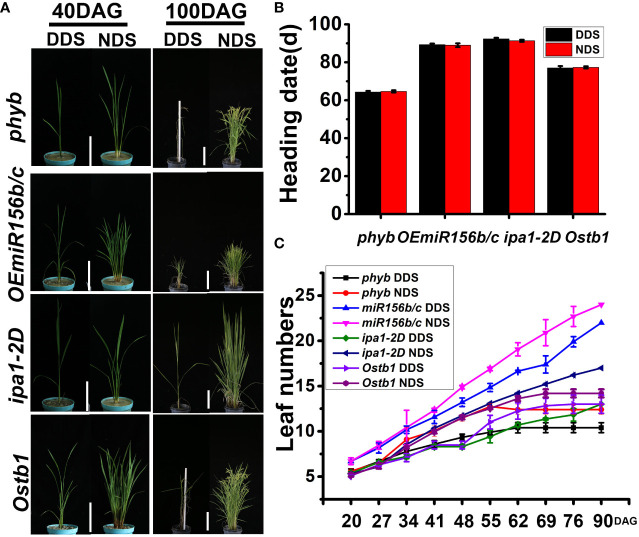
**(A)** A comparison of four mutants (i.e., *phyb、OEmiR156b/c、ipa1-2D、Ostb1*) of the tiller at different stages under DDS or NDS conditions. 40, 100 DAG treatment were photographed. Bar = 15 cm (left) Bar = 20 cm (right). **(B)** The same heading dates for four mutants (i.e., *phyb、OEmiR156b/c、ipa1-2D、Ostb1*) were observed under DDS conditions. **(C)** A fewer leaves were observed under DDS compared to NDS conditions in the four mutants (i.e., *phyb、OEmiR156b/c、ipa1-2D、Ostb1*). Error bars represent mean ± SD (n = 3) and different letters indicate significant differences as indicated by Duncan’s multiple range test (p ≤ 0.05).

In rice, *miR172* acts as a significant regulator of developmental changes. Plants with high levels of *miR172d* have been found to activate the flowering promotion genes *Ehd1* and *Hd3a*. The expression of *Hd3a* has been found to be briefly up-regulated at 48 DAG, when developmental changes associated with DDS occur, resulting in flowering four days early. The up-regulation of *miR172s* under DDS conditions causes the expression of *SNB* and *IDS1* to drop for practically the entire experimental duration. Furthermore, when compared to NDS, *SE5, GHD7*, and *HD5* all showed lower expression under DDS conditions at 48 to 56 DAG, indicating that loss-of-function mutants for these genes flower early. At 48 to 56 DAG, *HD1* and *RFT1* had the reverse expression pattern. DDS may switch genes on or off by indirectly affecting *miR156* and *miR172* expression, according to these findings. Due to the adaptability of rice cultivars to different geographical locations and cropping seasons, great emphasis should be paid to uncovering the genetic and molecular mechanism of rice photoperiodic blooming under DDS conditions. Because there are so many predominant alleles, such as *HD1*, *Ehd1*, and others (Doi et al., 2004; Leng et al., 2020) in different locations, the degree of SAS resulting from DDS may vary. The flowering of four mutants (i.e., *phyb、OEmiR156b/c、ipa1-2D、Ostb1*) was different for WT(NPB) under DDS and NDS conditions (**Figure 9**), indicating these alleles may retard SAS. Future studies may discover density-tolerant alleles in flowering-suppression or flowering-promotion pathways. Chloroplast dysfunction causes yellow leaves in DDS plants at 70 DAG, due to a decreased pigment content and the down-regulation of genes associated with chlorophyll metabolism and chloroplast development as a result of DDS.

The yield of direct seeding is determined by the efficiency with which the plants use radiation and the capacity with which they intercept light. Plants display a degree of mutual shading under a high planting density (i.e., under DDS conditions). According to increasing evidence, the SAS regulatory module is highly conserved in plants, and many genes involved in the SAS pathway may have been selected or discarded during rice domestication or breeding for better plant architecture and agronomic performance. A reduction of SAS could allow for higher plant productivity at higher densities or higher yield at current densities. This will be accomplished through the selection of natural variants as well as the generation of SAS mutations involving the genes mentioned above *via* genome editing. Therefore, further research should be focused on the effect of DDS on the SAS genes mentioned above.

## 5 Conclusion

**Figure 10:**
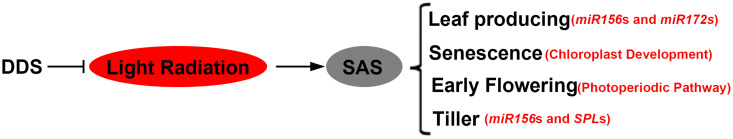
Simplified schematic model depicting the shade-avoidance response in DDS. Arrow: activate; Bar: repress.

We compared the responses of rice plants to NDS and DDS in this study. We proposed a putative DDS model based on our findings (**Figure 10**). In this model, DDS plants exhibit a constitutive SAS, in which solar radiation drops rapidly from the flag leaf to the base leaf, lowering phytochrome gene expression and changing the expression of multiple *miR156* or *miR172* genes. Because *miR156* is a conservative regulator of developmental timing in plants, a mutation in the *miR156* or *miR172* gene causes the expression of photoperiod-related genes to be misregulated, resulting in early flowering. Furthermore, DDS causes senescence by downregulating the expression of chloroplast synthesis-related genes throughout almost the entire stage. As a result, we hypothesized that predominant alleles (such as *HD1*, *Ehd1*, and so on) in different geographical regions may exhibit different degrees of SAS under DDS. In the future, density tolerant alleles could be found for the SAS-suppression or SAS-promotion pathways.

## Data availability statement

The original contributions presented in the study are included in the article/supplementary material. Further inquiries can be directed to the corresponding authors.

## Author contributions

YTC, LBG conceived the project and designed the research. YTC, MHZ, JS, HHF, JYW and JJW performed the qRT-PCR assay. YTC, MHZ, LBG performed miRNA northern blotting. JS, HHF, XZX and JJW performed phenotype observations. YTC wrote the article. All authors contributed to the article and approved the submitted version.
