# Supplementary material for: Expression dynamics of phytochrome genes for the shade-avoidance response in densely direct-seeding rice
Source: Front Plant Sci. 2023 Jan 18;13:1105882. doi: 10.3389/fpls.2022.1105882 (PMC9889870; doi:10.3389/fpls.2022.1105882)
Supplement: Supplementary file 1 [file Table_1.docx]

Supplemental Table1: Primers used in this paper.

| Primer name | Primers sequence (5'-3') |
| --- | --- |
| PhyA-RT-F | AGTTCTCTCCTGTTGGGGGT |
| PhyA-RT-R | CCCTGGTGCTTGATCCTAAGT |
| PhyB-RT-F | ATGGAACAGACACAATGCTT |
| PhyB-RT-R | AGCATACACCATATCAGCTT |
| PhyC-RT-F | ACAGCAACTGCGCCTATCTT |
| PhyC-RT-R | ATCCAGTGAGTTCTGCAGCC |
| miR156d-RT-F | GCATGGAGATGCTGCCAGTA |
| miR156d-RT-R | GATCCGGTTAATCCTGTTGATGA |
| miR156h-RT-F | AGCTGCTGCCTAGCTCCAGTA |
| miR156h-RT-R | TTCTCGGATCCGGTGAGAGA |
| miR172d-RT-F | GCAGCACCATCAAGATTCACA |
| miR172d-RT-R | CACATATAGTCAGCCAACCTCGAT |
| SE5-RT-F | TCCTATCTGGAAGAGCTGGC |
| SE5-RT-R | GGACACTGGGCAGAGGTCAT |
| RFT1-RT-F | CACCGGCTAGCTTAACCTTCCTGAACATC |
| RFT1-RT-R | GCCGGCCATGTCAAATTAATAACC |
| Hd3a-RT-F | CACCTGCTGCATGCTCACTATCATC |
| Hd3a-RT-R | CATGAGAGACCTTAGCCTTGC |
| Ehd1-RT-F | TGCAAATGGCGCTTTTGAT |
| Ehd1-RT-R | \| ATATGTGCTGCCAAATGTTGCT 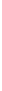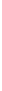 \| \| --- \| |
| Hd1-RT-F | TCAGCAACAGCATATCTTTCTCATCA |
| Hd1-RT-R | TCTGGAATTTGGCATATCTATCACC |
| Ghd7-RT-F | ATATTGTGGGAGCACGTT |
| Ghd7-RT-R | ATCTGAACCATTGTCCAAGC |
| Hd5-RT-F | GCTGCCTACTGATGCTGCTG |
| Hd5-RT-R | CTATCCCTCTTGGGGCTCTG |
| SNB-RT-F | ATGGAAGGGAAGCTGTTAC |
| SNB-RT-R | AATGTGGATGCTGGGACATC |
| OsIDS1-RT-F | CTGGCCTCCAGTTAACTTGT |
| OsIDS1-RT-R | GGCGCCGGCAGAGAATCCT |
| LchP2-RT-F | GAAGAAGATCAAGAACGGCC |
| LchP2-RT-R | TTGCCGGGGACGAAGTTGGT |
| OsCHLH-RT-F | GCACGGGAACTTGGCGTTTCATTA |
| OsCHLH-RT-R | ACATGTCCTGGAGCTGCTTCTCAT |
| OsCAO1-RT-F | TTGGCTCAGTTAATGAGGGCAGAATCC |
| OsCAO1-RT-R | \| GGATGCGCACGTTGAGCATCTTTGTGG 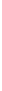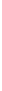 \| \| --- \| |
| OsDVR-RT-F | AGCCCAGGTTCATCAAGGT |
| OsDVR-RT-R | TGATCACCCTCTCGAAGAACT |
| OsHEMA-RT-F | GATGCAATCACTGCTGGAAAGCGT |
| OsHEMA-RT-R | CCATCTTGCCAGCACCAATCAACA |
| OsPORA-RT-F | ATGGCTCTCCAAGTTCAG |
| OsPORA-RT-R | TGGCTCACGCTAAGGAAC |
| OsPORB-RT-F | CCGCAAGGAGGGAGCGGTG |
| OsPORB-RT-R | CCCTCTTGGTGCTAAGGCCG |
| OsYGL1-RT-F | CCAGCCACTGATGAAAGCAGCAAT |
| OsYGL1-RT-R | AGAGCGCTAATACACTCGCGAACA |
| SPL3-RT-F | AACCAACGCGAAGGTCCTT |
| SPL3-RT-R | CCGGAAGCGACAGCAGAA |
| SPL13-RT-F | GCCGTTCCAGATCAGATAAACC |
| SPL13-RT-R | GCACGAACACACACACTCACACT |
| SPL14-RT-F | TAGCCATCATGCCCACTTC |
| SPL14-RT-R | AGACCAATCCATCGTGTTG |
| Ubiquitin F | AACCAGCTGAGGCCCAAGA |
| Ubiquitin R | ACGATTGATTTAACCAGTCCATGA |
